# Three-dimensional shape and deformation measurement on complex structure parts

**DOI:** 10.1038/s41598-022-11702-x

**Published:** 2022-05-11

**Authors:** Zhoujie Wu, Wenbo Guo, Zhengdong Chen, Haoran Wang, Xunren Li, Qican Zhang

**Affiliations:** College of Electronics and Information Engineering, Sichuan University, Chengdu, 610065 China

**Keywords:** Imaging and sensing, Imaging techniques

## Abstract

Stereo digital image correlation technique (stereo-DIC or 3D-DIC) has been widely used in three-dimensional (3D) shape and deformation measurement due to its high accuracy and flexibility. But it is a tough task for it to deal with complex structure components because of the severe perspective distortion in two views. This paper seeks to resolve this issue using a single-camera system based on DIC-assisted fringe projection profilometry (FPP). A pixel-wise and complete 3D geometry of complex structures can be reconstructed using the robust and efficient Gray-coded method based on a FPP system. And then, DIC is just used to perform the temporal matching and complete full-field pixel-to-pixel tracking. The in- and out-of-plane deformation are obtained at the same time by directly comparing the accurate and complete 3D data of each corresponding pixel. Speckle pattern design and fringe denoising methods are carefully compared and chosen to simultaneously guarantee the measuring accuracy of 3D shape and deformation. Experimental results demonstrate the proposed method is an effective means to achieve full-field 3D shape and deformation measurement on complex parts, such as honeycomb structure and braided composite tube, which are challenging and even impossible for the traditional stereo-DIC method.

## Introduction

Understanding the dynamic behavior of a structural component is essential to analyze its mechanical properties and prevent critical operating working conditions. The dynamic response of structural parts is required to test the structure parameters, guide the structural design and finally enhance their performance no matter in traditional manufacture (e.g., computer numerically controlled machine and aerospace manufacture) and advanced manufacture (e.g., additive manufacture and biofabrication)^1–3^. Conventional measuring methods use contact sensors (e.g., coordinate measuring machine and strain gagues) to achieve pointwise measurement and only acquire the information of several discrete points, which is hard to accurately describe the transient state and analyze the structural change of complex structure parts. Therefore, full-field and non-contact shape and deformation analysis for complex parts are urgently required to provide their corresponding quantitative characteristics.

Digital image correlation (DIC) has been proved to be a powerful non-contact technique for deformation measurement^4,5^. And it has been employed in many fields due to its multi-scale, non-contact and full-field measurement^6,7^. Recently, with the development of binocular stereo vision and high-speed photography technology, stereo digital image correlation (stereo-DIC or 3D-DIC) has been widely applied in dynamic 3D shape and deformation measurements^8–10^. However, the use of two high-speed cameras considerably increase the cost of measuring system and the precise synchronization of two high-speed camera is also a tough task^11^. To address the mentioned limitation in stereo-DIC, the grating-based^12^, prism-based^13^ and mirror-based^14^ single-camera stereo-DIC methods were proposed successively. These methods used diffraction grating, bi-prism lens and four-mirror adaptor to convert a single camera into two or three virtual cameras, which view a specimen from different views^15^. And all above strategies belong to optical splitting methods using one camera sensor to record two or more images, so less than half sensor is used to capture region of interest (ROI), reducing the spatial resolution of the measured results. To utilize full resolution of a camera, Pan proposed a high-speed stereo-DIC method using a color high-speed camera^16^ and full frames from different views can be recorded and retrieved by beam splitter system and color separation. And this kind of method needs to perform color cross-talk correction. In addition, for all stereo-DIC techniques, only the data in the overlapping field of view of two cameras can be used to calculate the shape and deformation, and the subset similarity between two views should be guaranteed for convergence of iterative algorithm. Therefore, this method is usually applied to measure the flat or curve surfaces. But for complex structure components, it is different to perform full-field 3D shape and deformation measurement because of large deformation between the subsets in left and right views caused by relative rotation between two cameras and complex shape of the tested specimen. However, in the fields of aerospace, intelligent manufacturing and material analysis, there are a lot of demands on shape measurement and deformation analysis of complex components, such as honeycomb structure, engine turbine, laminated structure^17–19^. And the measurement results of complex components in dynamic process can be used to analyze the structural performance and optimize the material parameters. So, it is required to find a technique to solve this problem and meet this requirement.

Fringe projection profilometry (FPP) has been well-studied for 3D shape measurement due to its high accuracy, low computation complexity and flexibility^20,21^. In FPP, pixel-wise phase-shifting algorithm can be chosen to retrieve shape information^22^, so it has advantages in the integrity of shape reconstruction and in the computation complexity for complex components compared with area-based correlation algorithms in DIC. Furthermore, with booming high-speed projecting and imaging equipment, dynamic 3D shape measurement techniques based on FPP have been rapidly developed in recent years^23–26^. Only one high-speed camera and one digital light processing (DLP) projector are required to accurately reconstruct shape information of dynamic scenes with binary defocusing technique^27^, which is low-cost compared with two expensive high-speed cameras in stereo-DIC system. Furthermore, binary defocusing technique can loosen the precise synchronization between high-speed camera and projector^28^, so it could be easily guaranteed by a simple trigger signal. However, the principle of fringe projection technique is projecting rather than attaching encoding information on tested surfaces, which makes it hard and even impossible to accurately track motion and deformation of the corresponding point. Therefore, the remaining task for FPP is to complete accurate deformation analysis.

To take full advantage of each technique, combination of FPP and DIC has been studied. Single-shot based and multiple-shot based methods have been researched depending on different applicable scenes. Only one image is required in single-shot methods to reconstruct one 3D data, so it is suitable for measuring high-speed transient scene. Fourier transform profilometry (FTP) is one of the representative single-shot reconstruction methods, in which spectrum filter or color channel separation is used to extract texture map from a captured speckle-embedded fringe pattern^29^. But the filtering operation limits the reconstruction precision of FTP, making it sensitive to nonuniform reflectivity and hard to restore sharp edges and abrupt change. In addition, no additional assisted information can be obtained in the single-shot method, so spatial phase unwrapping algorithms are usually used to unwrap the wrapped phase, leading to the phase uncertainty for the isolated objects. For the multiple-shot methods, 3D reconstruction is achieved in the temporal domain and it can be independently calculated for each pixel, so this kind of method is suitable to restore discontinuous or colored surface. Phase measuring profilometry (PMP) is a well-known multiple-shot method to accurately reconstruct the shape of the textured surfaces and temporal phase unwrapping (TPU) technique can be used to eliminate phase ambiguity of discontinuous surfaces^30^. In the reported combining methods, single-shot methods are used to measure continuous surfaces such as a bending surface^31^, impacted bonnet^32^ and aluminium plate^33^ and multiple-shot methods achieve measurement of flat section of the cushion made of molded pulp product^34^, planar specimen with a hole and a step^35^ and rotating blades^36^. All the mentioned measured scenes are relatively flat and could also be completed using stereo-DIC techniques. But to the best of our knowledge, there are no report on shape and deformation measurement of complex components using combining methods.

In this work, a one-camera and one-projector system based on DIC-assisted fringe projection profilometry is used to achieve 3D shape measurement and deformation analysis on complex structure parts. Specifically, first a single-camera measurement system is developed using the high-robust and high-efficient 3D shape measurement method based on Gray-coded light, in which a pixel-wise and complete 3D geometry of complex structures can be reconstructed using every four projected patterns. Then, speckle pattern fabrication and fringe denoising methods are carefully compared and chosen to simultaneously guarantee measuring accuracy of 3D shape and deformation. And finally, DIC method is just used to match the corresponding point at different time instead of solving the in-plane displacement, and the 3D deformation analysis is performed by comparing the accurate and complete 3D data of each corresponding pixel. And the presented experimental results demonstrate the presented method is able to realize 3D shape and deformation measurement on complex parts, which is a hard task for the traditional stereo-DIC method.

## Methods

**Figure 1:**
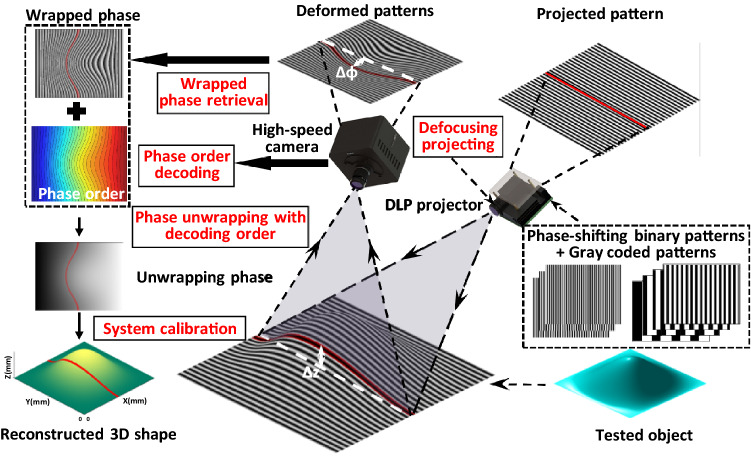
Schematic diagram of a FPP system for 3D shape measurement.

The typical 3D shape measurement system based on FPP is shown in Fig. 1. Firstly, phase-shifting sinusoidal fringes and encoding patterns (Gray coded patterns in this work) are projected to modulate the height of the measured surface. Then, the camera captures the deformed patterns from another angle. Next, wrapped phase can be retrieved from phase-shifting patterns and the phase order can be decoded from Gray coded patterns. And finally, the 3D shape can be reconstructed after phase unwrapping and system calibration.

### Robust and efficient 3D shape measurement based on Gray-coded-based method

It is contradictory for the requirement of surface texture to FPP and DIC. FPP prefers uniform surface for high fringe contrast while DIC demands textured surface with significant intensity difference. Therefore, rich texture is treated as beneficial mark for deformation analysis using DIC but unexpected noise for shape measurement using FPP. To weaken this contradiction, robust shape measurement method should be applied.

Gray coding is a robust binary coding strategy because there is only one hamming distance between two adjacent decoding words. So, it is often used to resist strong noise^37–39^. In this work, complementary Gray code method^40^ is adopted to ensure the robust phase unwrapping.

**Figure 2:**
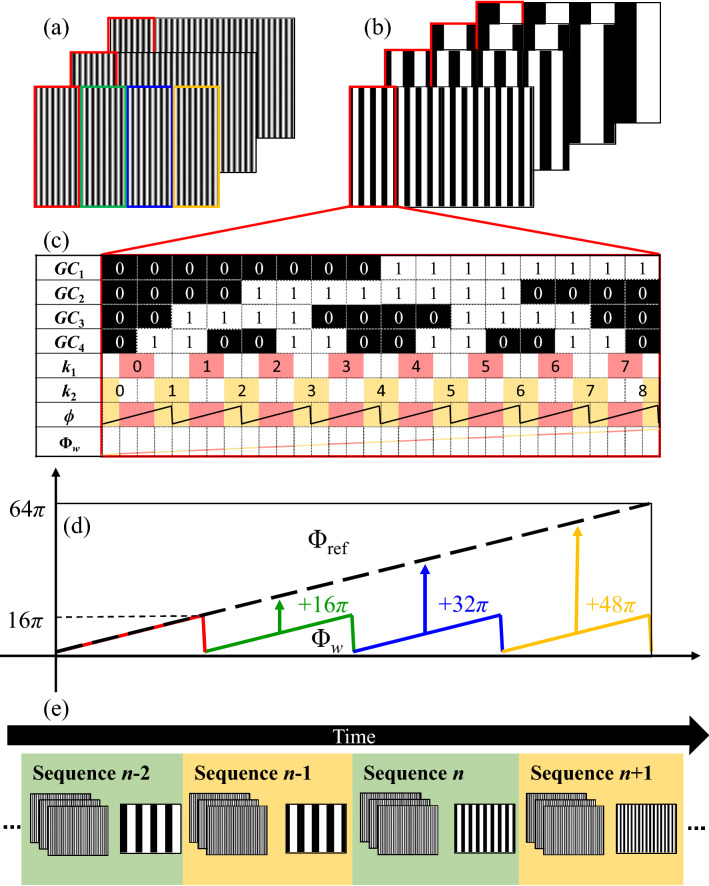
Principle of the robust and efficient 3D shape measurement based on Gray-coded-based method. (**a**) Projected phase shifting patterns. (**b**) Projected Gray code patterns. (**c**) Decoding process using complementary strategy. (**d**) Period extension based on reference phase. (**e**) Efficiency improvement using time-overlapping coding strategy.

As shown in Fig. 2a,b, binary dithering technique^27^ and three-step phase-shifting algorithm are used to produce and project three binary phase-shifting pseudo-sinusoidal patterns with a high switching rate to the tested object. And the high-quality sinusoidal patterns can be generated on the image plane of the used camera, which are described as:

1 $$I_{1} (x,y) = \alpha (x,y)\left\{ {a^{p} + b^{p} \cos [\phi (x,y) - 2\pi /3]} \right\}$$

2 $$I_{2} (x,y) = \alpha (x,y)\left\{ {a^{p} + b^{p} \cos [\phi (x,y)]} \right\}$$

3 $$I_{3} (x,y) = \alpha (x,y)\left\{ {a^{p} + b^{p} \cos [\phi (x,y) + 2\pi /3]} \right\}$$

In which *a*^*p*^ and *b*^*p*^ are the mean value and the modulation of sinusoidal fringe pattern generated in projector space; *ϕ*(*x*, *y*) is the wrapped phase of the deformed fringe patterns and solved using Eqs. (1)–(3).

4 $$\phi (x,y) = \tan^{ - 1} \frac{{\sqrt 3 (I_{1} (x,y) - I_{3} (x,y))}}{{2I_{2} (x,y) - I_{1} (x,y) - I_{3} (x,y)}}$$

The arctangent operation causes the phase ambiguity, so Gray-coded patterns are generated to uniquely encode the fringe order and unwrap the wrapped phase. In the traditional Gray-coded method, *N* Gray-coded patterns can label maximum 2^*N*^ fringe orders. But jump errors easily occur on the boundaries of white and black codes due to the defocus of projector and the motion of object. So, an additional Gray code pattern with half period of fringe pattern is projected in the complementary Gray code method to avoid jump errors. The decoding process is shown in Fig. 2c, and the encoding period is 8 in this example. After capturing and binarizing Gray coded patterns, the phase order *k*_1_(*x*, *y*) can be calculated using traditional *N* patterns:

5 $$V_{1} (x,y) = \sum\limits_{i = 1}^{N} {GC_{i} (x,y) \times 2^{(N - i)} } ,$$

6 $$k_{1} (x,y) = LUT(V(x,y)),$$

in which, *GC*_*i*_(*x*, *y*) denotes the *i*_th_ binarized Gray-coded pattern, *V*_1_(*x*, *y*) is the decoding decimal number and the function *LUT*(∙) is used to look up the known unique relationship between *V*_1_(*x*, *y*) and *k*_1_(*x*, *y*). And the phase order *k*_2_(*x*, *y*) can be solved using all *N* + 1 patterns:

7 $$V_{2} (x,y) = \sum\limits_{i = 1}^{N + 1} {GC_{i} (x,y) \times 2^{(N + 1 - i)} } ,$$

8 $$k_{2} (x,y) = INT((LUT(V_{2} (x,y)) + 1)/2).$$

In which, function *INT*(∙) returns the nearest integer downward. It can be seen from Fig. 2c, the edges of *k*_2_(*x*, *y*) are shifted half period with that of *k*_1_(*x*, *y*). So, the different regions of wrapped phase can be correctly unwrapped using their middle regions (labeled in red and yellow) of the corresponding phase orders which have no words changes:

9 $$\Phi _{w} (x,y) = \left\{ {\begin{array}{*{20}l} {\phi (x,y) + 2\pi k_{2} (x,y),} \hfill & {\phi (x,y) \le - \pi /2} \hfill \\ {\phi (x,y) + 2\pi k_{1} (x,y),} \hfill & { - \pi /2 < \phi (x,y) < \pi /2} \hfill \\ {\phi (x,y) + 2\pi k_{2} (x,y) - 2\pi,} \hfill & {{\mkern 1mu} \phi (x,y) \ge \pi /2} \hfill \\ \end{array} } \right.$$

In which, *Φ*_*w*_(*x*,*y*) is the initial unwrapping phase in subregions. And using this strategy, edge errors can be avoided and robust phase unwrapping can be achieved.

For the traditional Gray code method, denser fringe period means higher measuring accuracy but it increases the number of projected Gray code pattern and decreases the measuring efficiency. In order to simultaneously guarantee the measuring accuracy and efficiency in dynamic measurement, we introduce the period extension method based on reference phase and time-overlapping coding strategy into complementary Gray code method in this paper.

As shown in the red rectangle of Fig. 2a,b, fringe patterns with 8 periods are encoded using three traditional Gray code patterns and one complementary Gray code pattern. And all the patterns are copied four times to extend the encoding periods. Each part of wrapped phase can be unwrapped using Eq. (4) as shown in Fig. 2d and only 4 discontinuities with large phase jump (16π) remain. The unwrapping phase of reference phase Ф_ref_(*x*,*y*) which is obtained in calibration process is introduced to assist in eliminating the remained phase ambiguity using:

10 $$\Phi (x,y) = \Phi_{w} (x,y) + 16\pi \times round[\frac{{\Phi_{ref} (x,y) - \phi_{w} (x,y)}}{16\pi }].$$

Actually, there is a successful condition of this method to ensure the phase difference caused by modulation of object within 16*π*, which can be easily satisfied in actual measurement. Thus, the encoding period can be extended to 32 to improve measuring accuracy using 4 Gray code patterns.

To further improve the measuring efficiency, each traditional Gray coded pattern is projected following consecutive three binary sinusoidal patterns as shown in Fig. 2e. For each group of sinusoidal patterns, 4 Gray-coded patterns, which are close to sinusoidal patterns, are used to unwrap the wrapped phase. Therefore, every Gray-coded pattern is reused four times to reduce the projected number in each projecting sequence from seven to four.

### Speckle pattern separation and fringe denoising

In dynamic measurement, fringe patterns and texture maps are expected to be obtained at the same time to ensure the consistency of the measured shape and deformation. However, high-quality speckle pattern is the promise of accurate deformation measurement but is treated as noise for shape measurement using FPP, so high-quality speckle pattern separation from fringe patterns and fringe denoising should be executed for accurate shape and deformation measurement, respectively.

For deformation measurement, a stable and contrast speckle pattern is required. So, the modulation of the obtained sinusoidal patterns which is immune to varying ambient light, is used to obtain the texture image:

11 $$M(x,y) = \frac{2}{3}\sqrt {\left[\sum\limits_{i = 1}^{3} {I_{i} (x,y)} \sin (2\pi i/3)\right]^{2} + {\left[\sum\limits_{i = 1}^{3} {I_{i} (x,y)} \cos (2\pi i/3)\right]^{2} .}}$$

Substituting Eqs. (1)–(3) into Eq. (11):

12 $$M(x,y) = b^{p} \alpha (x,y)$$

For shape measurement, the introduced Gray-coded-based method can ensure the robust phase unwrapping, but the influence of rich texture on measuring accuracy of FPP cannot be ignored. So, it is highly desirable to eliminate or minimize the effect of noise before applying the fringe pattern for measurement^41^. Therefore, how to design the speckle pattern and perform appropriate fringe denoising process becomes a crucial step before 3D reconstruction.

**Figure 3:**
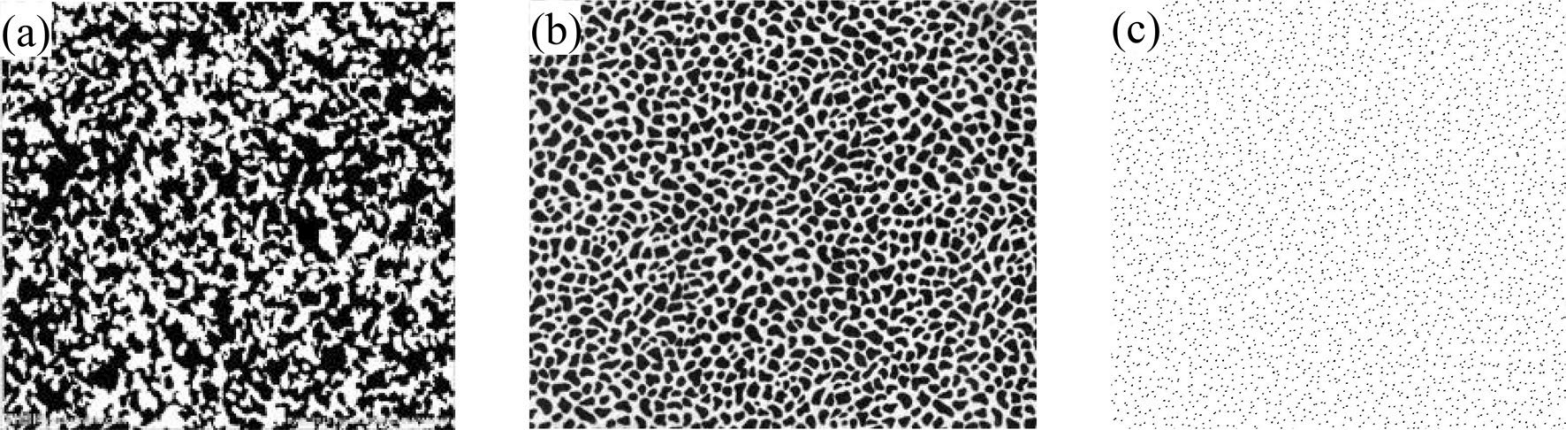
Three typical speckle patterns.

Three typical speckle patterns are shown in Fig. 3. In the dark areas of the speckle pattern shown in Fig. 3a,b, signal-to-noise ratio of the fringe pattern is low, which will cause large errors in the reconstructed result. But the speckle pattern can be treated as random noise when it is discrete distributed as shown in Fig. 3c, the fringe denoising method can be applied to eliminate noise from fringe patterns. So, speckle patterns with discrete distribution in Fig. 3c is preferred in the combining methods of FPP and DIC. And median filter is mainly useful to remove the impulse noise and simultaneously preserve image details, so it is applied to achieve fringe denoising in this work. And the comparison on performance of different speckle patterns is conducted in Sec. 3.1.

### 3D deformation measurement by feature-assisted digital image correlation

Accurate 3D shape data can be obtained from FPP system and the high-quality texture map is extracted from the fringe patterns. Thus, the next step is to track the corresponding points at different states. As a powerful full-field analysis method, DIC is widely used to perform displacement and deformation analysis based on the similarity of the image intensity distribution before and after deformation. So, it is adopted to achieve accurate and pixel-wise image tracking. For a given point *P*(*x*_0_, *y*_0_) in the reference image, the widely used zero-mean normalized cross correlation (ZNCC) is used to determine the position and shape of the subset in the deformed image by maximizing a correlation coefficient^42^:

13 $$C_{ZNCC} ({\mathbf{p}}) = \frac{{\sum {_{\Omega } \left( {F(x,y) - \overline{F} } \right)\left( {G(x^{*} ,y^{*} ) - \overline{G} } \right)} }}{{\sqrt {\sum {_{\Omega } \left( {F(x,y) - \overline{F} } \right)^{2} } } \sqrt {\sum {_{\Omega } \left( {G(x^{*} ,y^{*} ) - \overline{G} } \right)^{2} } } }},$$

in which, Ω is the selected subset; *F*(*x*, *y*) and *G*(x^*^, y^*^) denotes the intensity distribution of the reference and deformed images; $\overline{F }$ and $\overline{G }$ are the mean intensities in specific subsets. And **p = **(*u*, *v*, *u*_*x*_, *u*_*y*_, *v*_*x*_, *v*_*y*_) is the deformation parameter to determine the shape function which describes the subset’s shape change. And the common first-order shape function is used^43^ to update the subset using the advanced inverse-compositional Gauss–Newton (IC-GN) algorithm for subpixel registration^44^:

14 $$\left\{ {\begin{array}{*{20}c} {x^{*} = x + u + u_{x} (x - x_{0} ) + u_{y} (y - y_{0} )} \\ {y^{*} = y + v + v_{x} (x - x_{0} ) + v_{y} (y - y_{0} )} \\ \end{array} } \right..$$

For the measurement of complex parts, obvious discontinuous surfaces and large deformation make it difficult or even unsuccessful in initial guess. So, scale-invariant feature transform (SIFT)^45^ is used to obtain a convergent initial guess for complex parts.

It should be mentioned that in FPP measuring system, all the deformed patterns are captured from one view, and experimental study indicates that the IC-GN algorithm with first-order shape function suffices for the temporal match in most cases of deformation measurement in engineering application^5^ whereas the IC-GN algorithm with second-order shape function is more suitable for stereo matching. Therefore, in this proposed DIC-assisted FPP system, the time-consuming update with the second-order shape function can be omitted.

After obtaining pixel-wise matching results, the in- and out-of-plane deformation are simultaneously obtained by subtracting 3D data of the matching point, which is obtained by FPP with sub-pixel bicubic interpolation, using Eq. (15).

15 $$\left\{ \begin{gathered} U(x,y) = X(x^{*} ,y^{*} ) - X(x,y) \hfill \\ V(x,y) = Y(x^{*} ,y^{*} ) - Y(x,y) \hfill \\ W(x,y) = Z(x^{*} ,y^{*} ) - Z(x,y) \hfill \\ \end{gathered} \right.$$

## Results

Our measuring systems was developed with a high-speed camera (Photron FASTCAM Mini AX200) and a high-speed projector (DLP VisionFly6500). The lens embedded in the used camera has a focal length of 16 mm and an aperture of f/1.4 and the resolution of the projector is 1920 × 1080 pixels. In all dynamic experiments, the synchronized image refreshing rate of the projector and capturing rate of camera was set at 4000 Hz, and the camera resolution was set at 1024 × 1024 pixels. In addition, the period of the projected sinusoidal fringe patterns is set as 32, which is increased from 8 to 32 with help of the reference phase. It should be mentioned that the measuring depth range of this DIC-assisted FPP system depends on the overlapping range for effective depth of focus of the projector and the camera.

**Figure 4:**
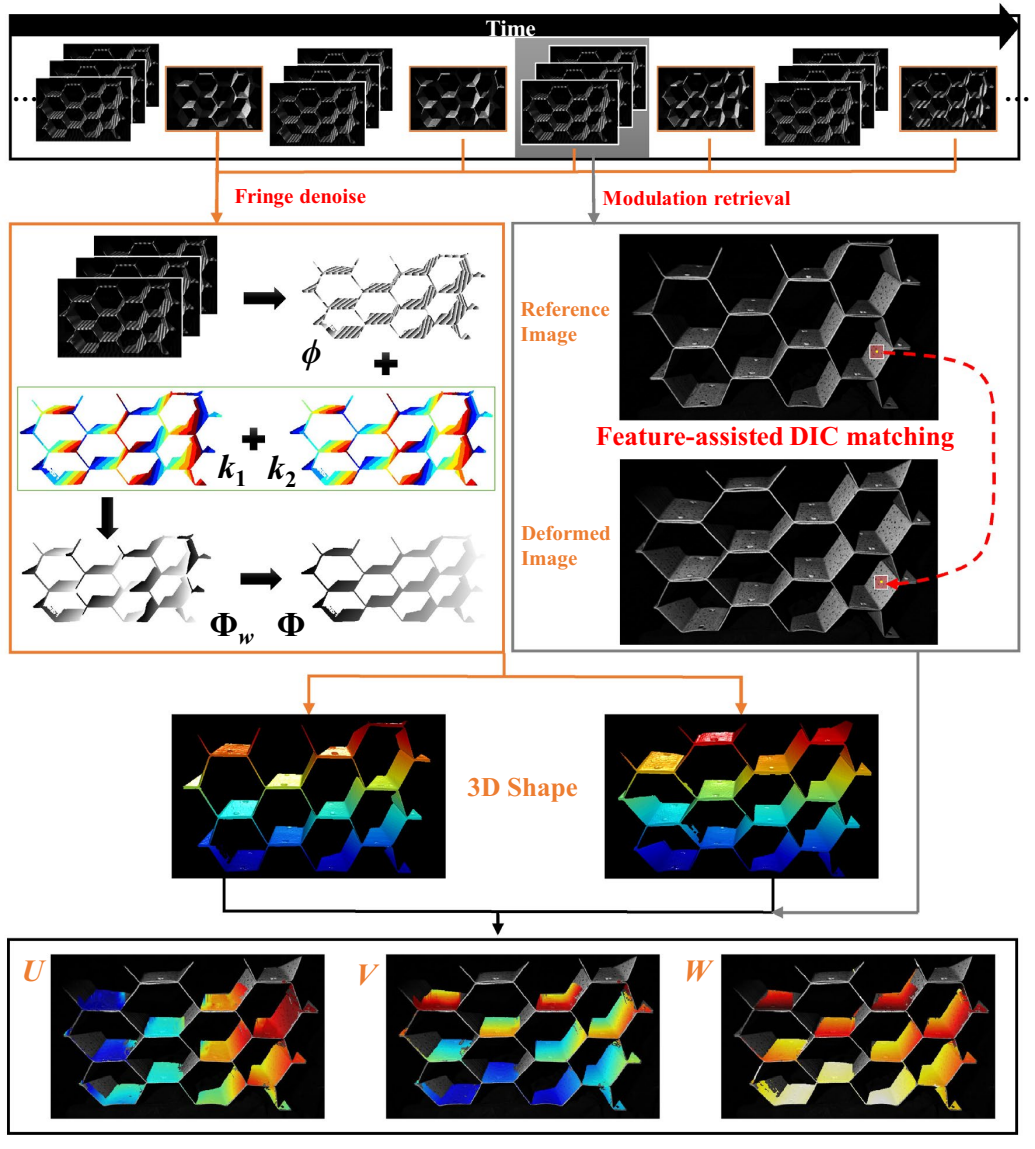
Framework of the proposed 3D shape and deformation measurement method.

The framework and intermediate results are shown in Fig. 4.

Firstly, the time-overlapping coding strategy is used to project the binary sinusoidal patterns and redesigned Gray coded patterns. After median filtering, the influence of speckle pattern on restored phase can be reduced and the robust phase unwrapping can be achieved using complementary phase order *k*_1_ and *k*_2_ and the reference phase. Next, the stable texture map is separated from three phase-shifting patterns. And the extracted texture map is used to perform corresponding point tracking using feature-assisted DIC algorithm. At last, the in- and out-of-plane deformation are calculated by comparing the accurate and complete 3D data of each corresponding point.

### Speckle pattern fabrication and assessment for shape and deformation measurement

**Figure 5:**
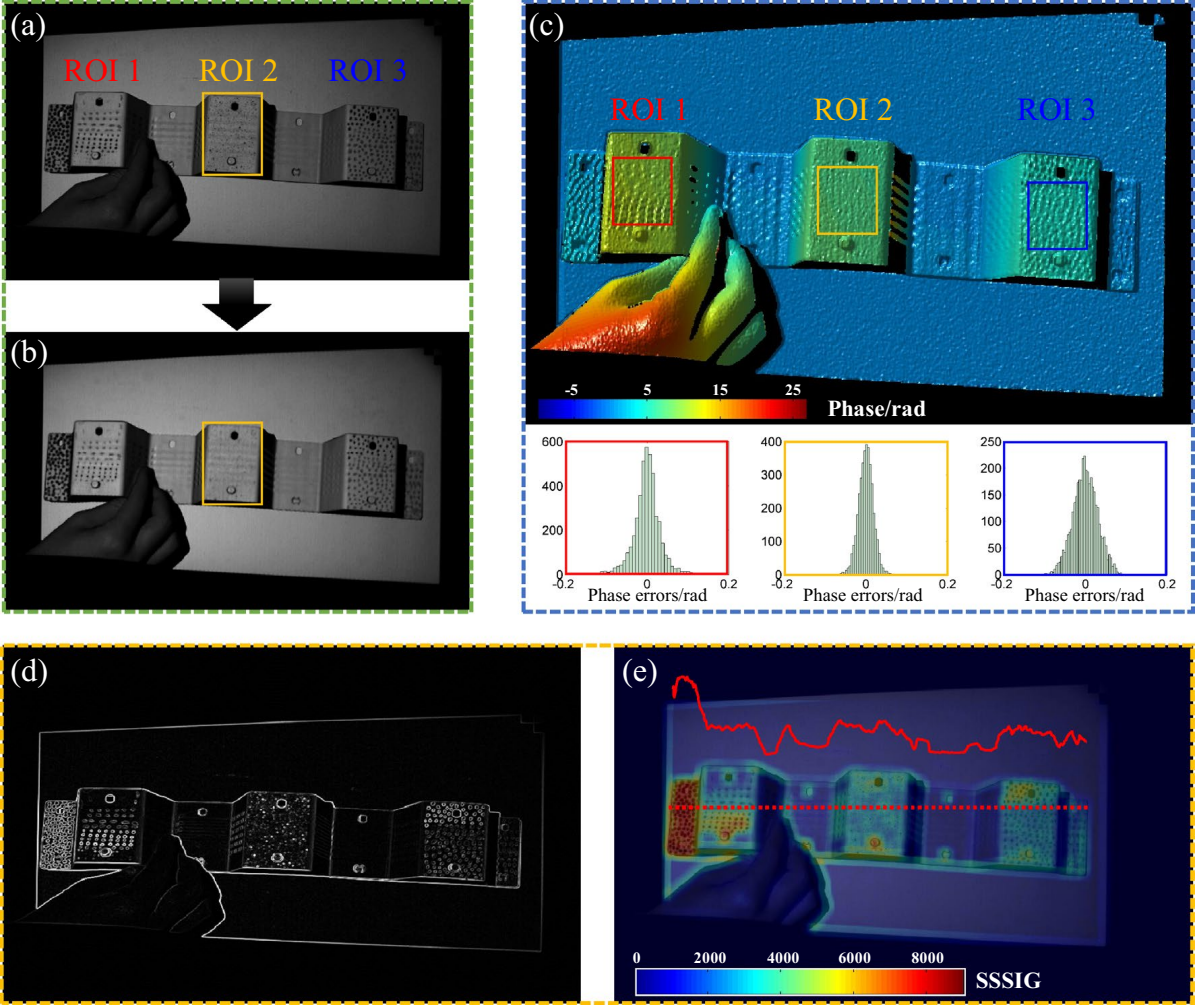
Comparative experiment on shape and deformation measurement accuracy using different speckle patterns. (**a**) Extracted texture pattern from phase-shifting patterns. (**b**) Texture pattern after median filtering. (**c**) Reconstructed phase and errors distribution in three regions. (**d**) Intensity gradients of tested specimen. (**e**) SSSIG of tested specimen.

To design the suitable speckle pattern to guarantee measurement accuracy of both shape and deformation, the experimental assessment is conducted. Three kinds of speckles are fabricated on three regions of the tested specimen as shown in Fig. 5a. The speckles on region of interest (ROI) 1 and ROI 3 are painted using a marking pen and a pencil, respectively, and the speckle is sprayed on ROI 2 using black paint. Figure 5a is the modulation of the original phase-shifting patterns and median filtering is applied in fringe patterns to reduce the influence of speckle on accurate phase retrieval. The modulation of phase-shifting patterns after filtering is shown in Fig. 5b and the corresponding reconstructed phase is shown in Fig. 5c.

Results show that the median filtering performs well to remove the discrete sprayed speckle while keeping the details of the specimen but the gathering painted speckle cannot be removed from fringes. And the reconstructed phase using filtering fringes and error distribution on three regions are given in Fig. 5c, which shows phase on ROI 2 with discrete sprayed speckle has the minimum error. In addition, speckle quality is also our concern to determine the deformation measurement accuracy and the sum of square of subset intensity gradients (SSSIG) can be used to accurately predict deformation accuracy as proved in^46^. So, the intensity gradient and SSSIG with the size of subset 21 × 21 pixels are calculated and shown in Fig. 5d,e. It can be found that speckle quality of ROI 2 before filtering is slightly lower than that of ROI 1 but higher than that of ROI 3, which can ensure the matching accuracy in deformation measurement. All results demonstrate that the sprayed discrete speckle shown in Fig. 3c and ROI 2 is suitable to be used in the DIC-assisted FPP system to guarantee the accuracy of 3D shape and deformation at the same time. So, the spraying method is adopted in our next experiments.

### Comparison of reconstructed integrity and computational efficiency

**Figure 6:**
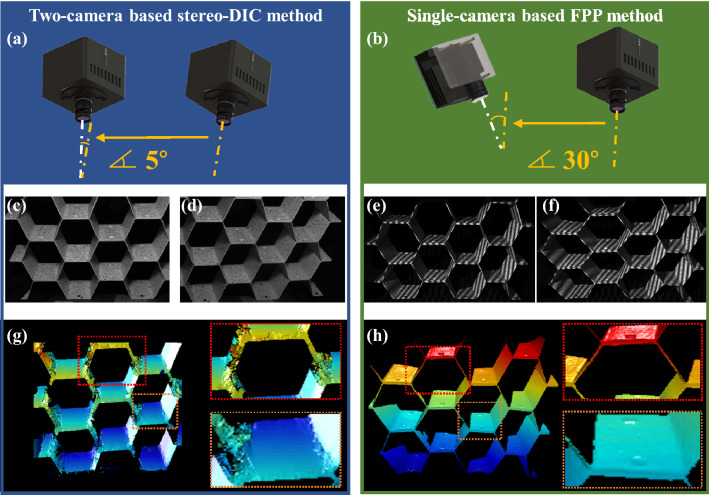
Comparative experiment on reconstruction of a complex honeycomb structure using stereo-DIC and FPP. (**a**) Measuring equipment of two-camera based stereo-DIC method. (**b**) Measuring equipment of single-camera based FPP method. (**c**, **d**) Captured images from left and right cameras in stereo-DIC. (**e**, **f**) Captured fringe patterns at two moments in FPP. (**g**) Reconstructed disparity map in stereo-DIC. (**h**) Reconstructed depth map in FPP.

To verify the effectiveness and superiority of the proposed DIC-assisted FPP measurement system on complex parts compared with the traditional stereo-DIC measurement system, the comparative experiments on honeycomb structure using two methods are performed. The measured setups of two methods are given in Fig. 6a,b. For this particular complex structure with deep hole and sharp edges, the angles between two equipment are set as an optimal angle for two different systems on the precondition of successful reconstruction.

For the stereo-DIC method, it is used to match image pairs deriving from an optical stereo setup (with a typical angle of 30°)^47^, but for the traditional DIC algorithm, it is difficult to match images when their relative rotation exceeds 7°^48^. This is because sub-pixel iteration algorithm such as IC-GN algorithm needs a fine initial guess to guarantee the convergence. The introduced SIFT algorithm can be used for this purpose, but it does not perform well when there is severe perspective distortion since the corresponding features appear significantly different in the images of two perspectives^49^. Furthermore, if the tested specimen has complex structures such as deep hole, sharp edges, thin wall, high curvature regions or concave-convex structure, it becomes more challenging for stereo-DIC. Therefore, in our experiment, we adjusted the angle between two cameras to 5° to ensure the success of stereo-DIC on this complex specimen and the captured images and reconstructed disparity map are shown in Fig. 6c,d,g. Because stereo-DIC is the area-based method, it is unable to reconstruct the information of sharp edges and loses the details as shown in Fig. 6g.

For the presented DIC-assisted FPP system, the camera is put in the same position with that in the stereo-DIC method but the angle between the projector and camera is set as 30° for higher measuring accuracy. Phase retrieval and 3D reconstruction are calculated pixel-by-pixel, therefore 3D data of all pixels which are illuminated by the projector and captured by the camera can be reconstructed. And the measuring principle guarantees that measurement result of FPP system is immune of severe perspective distortion and sharp edges. In addition, the projected lens usually has larger aperture and divergent angle compared with that of cameras, so the FPP system performs well with a relatively large angle between the camera and projector for higher measuring accuracy. The captured deformed fringe patterns at two moments and the reconstructed depth at second moment are shown in Fig. 6e,f,h. It can be noted that the integrity of the result can be ensured even in the sharp edges and the details like concave and convex in the specimen. Experimental results demonstrate the FPP system has advantages on measuring integrity and details preservation for 3D shape measurement.

**Figure 7:**
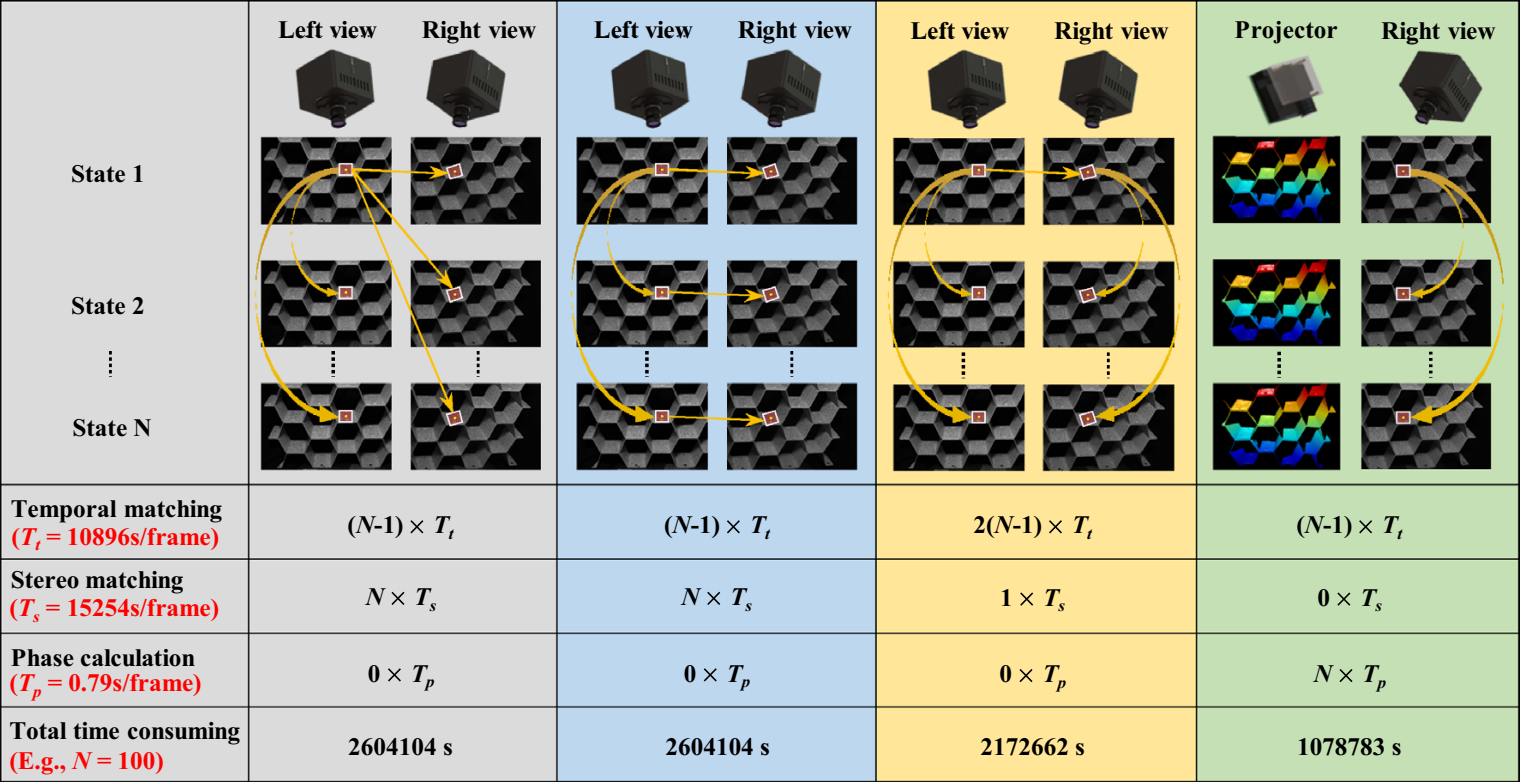
Comparison of computational efficiency for different strategies in stereo-DIC and proposed DIC-assisted FPP system.

Besides the reconstructed integrity, the computational cost is also concerned in dynamic reconstruction. For stereo-DIC method, stereo matching is used to reconstruct the 3D coordinates by triangulation method while temporal matching is applied to track the corresponding point and calculate the displacement or deformation. Due to the non-linear nature of the perspective distortion, second-order shape functions should be used for stereo matching which brings higher computational cost. But for the temporal matching, the IC-GN algorithm with first-order shape functions is still applicable in most cases because of the same view, which has higher computational efficiency^5^. Actually, three different matching strategies can be applied in stereo-DIC as shown in Fig. 7, in which the first one shown in first column is preferred because only one stereo matching is performed. But for this strategy, additional 2*N* − 2 temporal matching is still required whereas *N* − 1 temporal matching and *N* stereo matching is needed for other two strategies for stereo-DIC. For the FPP method, total *N* − 1 temporal matching is required without stereo matching and the 3D coordinates can be obtained from *N* simple phase calculation.

To compare the computational efficiency of these two types of methods, we counted the time of image matching in stereo-DIC method and phase calculation in FPP method for one frame. The IG-GN algorithm with first-order shape functions is applied for temporal matching; the IG-GN algorithm with second-order shape functions is applied for stereo matching; phase-shifting algorithm, complementary Gray code (CGC) decoding algorithm and period extension algorithm are used to calculate unambiguous phase. All the algorithms were implemented in MATLAB platform (Intel Core™ i5-8250U CPU with 1.60 GHz and DDR3 1333 MHz RAM with 8 GB) and the image resolution is 1024 × 1024 pixels. The subset size is set to 21 × 21 pixels and all pixels are performed using tracking algorithm and phase extracting algorithm. As shown in Fig. 7, the computational time of temporal matching for disparity, stereo matching for disparity and phase calculation for one frame is 10,896 s, 15,254 s and 0.79 s, respectively. If *N* is assumed as 100, the total computational cost of DIC-assisted FPP system is about half of stereo-DIC system.

It should be mentioned that the step size in DIC method is usually set as half of the subset size (10 pixels in this example) to avoid resultant calculation and interpolation algorithm is used to obtain a continuous deformation field in DIC community. And even this process is performed, the computational cost of phase calculation is much less than that of DIC matching and can be ignored. So, the computational cost of FPP system is much less than the stereo-DIC system with third efficient strategy in Fig. 7 for 3D shape and deformation measurement. Therefore, the FPP-based strategy has obvious advantage in computational efficiency compared with the traditional stereo-DIC strategy.

### Shape and deformation measurement on complex structure part

**Figure 8:**
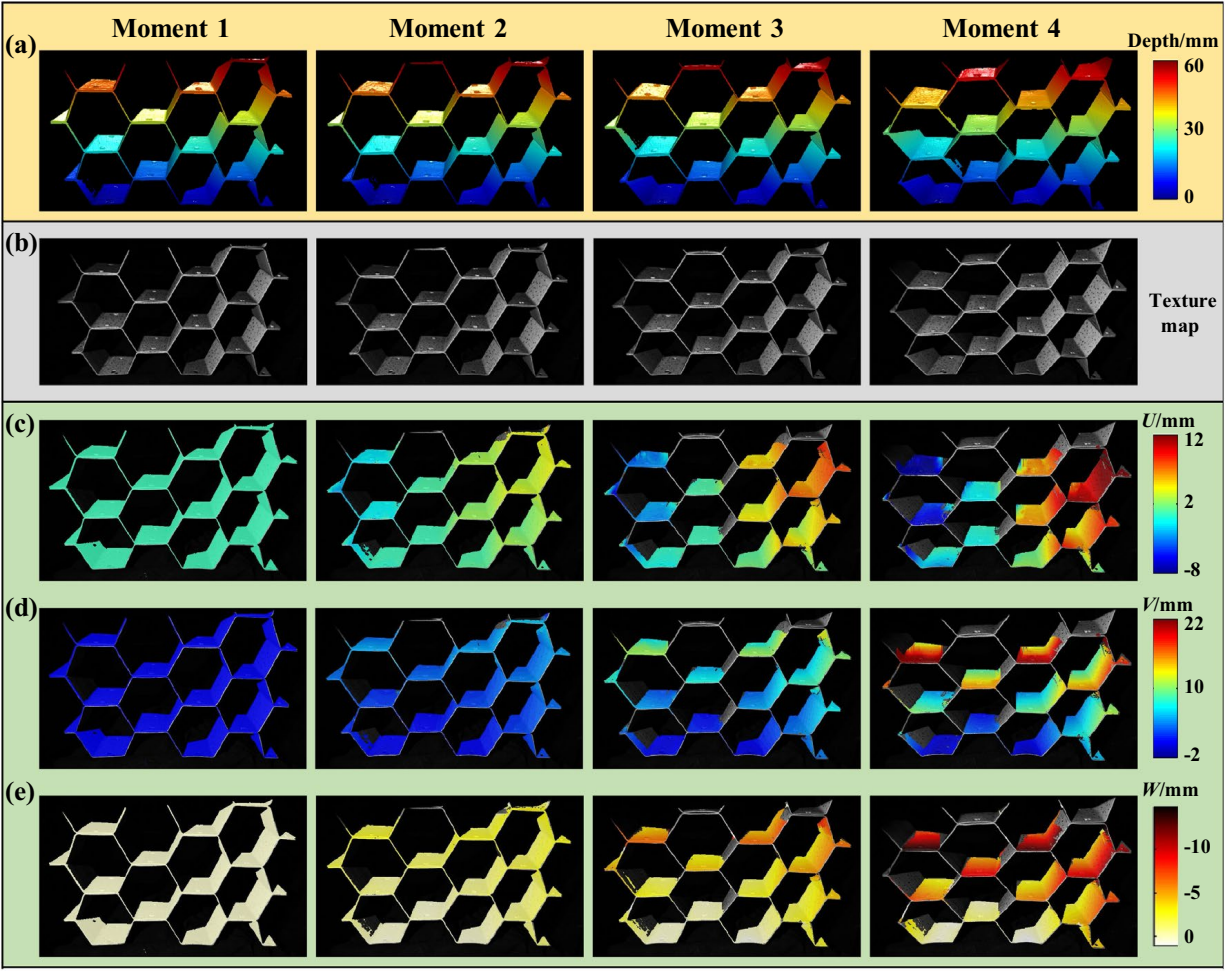
Measured results of the deformed honeycomb structure. (**a**) Reconstructed shapes at different moments. (**b**) Retrieved texture maps. (**c**–**e**) Restored *U*, *V*, *W* deformation at corresponding moments compared with that of moment 1.

Using the proposed DIC-assisted system, the deformation process of a complex part is captured and measured. The measured specimen is a honeycomb structure part, which is widely applied in aerospace manufacturing, material science and structural mechanics with its excellent geometrical and mechanical properties. To better understand the structural properties, complete and continuous 3D shape and deformation of this structure by the force is required. It is a challenge for the traditional non-contact method like stereo-DIC as described in last subsection. But with the advantage of the pixel-wise detection of the proposed DIC-assisted FPP, this complex structure can be measured and the reconstructed results are shown in Fig. 8. The measured part is loaded by oblique downward squeeze and the complete 3D shape results at four typical moments are shown in Fig. 8a; the extracted high-quality texture maps of corresponding moments are shown in Fig. 8b; and the 3D deformation are calculated by temporal matching and 3D coordinates subtracting as shown in Fig. 8c–e. Experimental data proves that the presented measuring method can realize pixel-wise 3D shape measurement and deformation analysis for complex structure parts.

## Discussion

Experiments have proved the effectiveness of the proposed single-camera DIC-assisted FPP measuring system on complex structure parts. Some potential applications of the proposed method are suggested as follows.

1. Application in special environment with distortions for better measurement stability. In some special application, DIC are performed using special imaging devices such as scanning electron microscope (SEM), which brings non-negligible image distortion and reduces measuring accuracy^50^. In SEM DIC, temporarily- and spatially-varying distortions are non-negligible in SEM image because of the fundamentally different imaging process in an SEM system from an optical system^51^. And the cumulative effect of both drift and spatial distortions can introduce large error in further displacement and strain analysis^52,53^. Time-varying distortion or drift distortion occurs throughout the scan process, which is non- stationary. For SEM DIC, an equivalent stereo-vision methodology is developed to mimic two cameras by tilting sample stages, so two obtained SEM images before and after tilting sample stages need distortion correction, respectively. But for the proposed single-camera system, only one image should be corrected. In addition, no mechanical movement in the proposed method, so the reconstruction error due to rotation angle variations in SEM DIC can also be avoided. Therefore, the proposed single-camera system is potential to be applied in special environment with distortions for better measurement stability once a microscopic projection unit being designed and embedded in SEM to illuminate the measuring field.
2. Application on complex parts with fine structure for higher resolution. It is commonly accepted that mechanical properties, such as strength and stiffness, are intrinsically dependent on the designed structure using different materials^54^. Therefore, high-resolution shape, deformation and strain of complex parts are expected to be measured. To demonstrate the potential high resolving power of the proposed method, a comparative experiment is performed on braided composite tubes^55,56^. A biaxially braided tube is weaved using braided straps with fine structure as shown in Fig. 9f.

**Figure 9:**
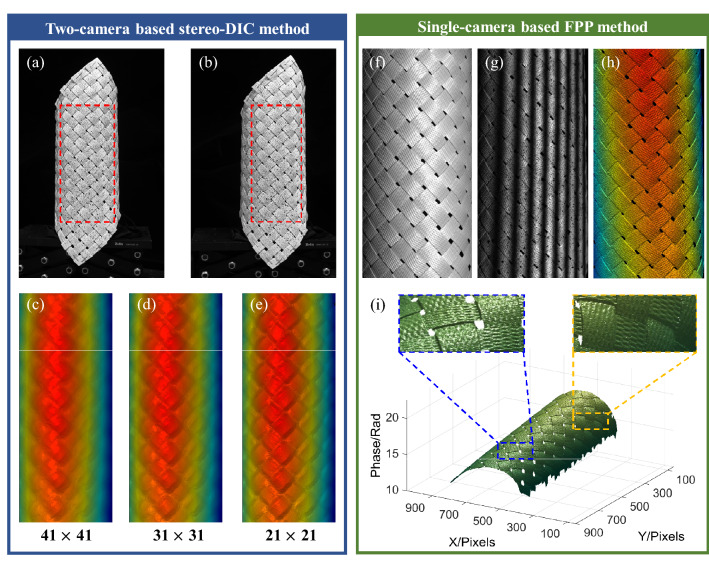
Comparative experiment on reconstruction of a biaxially braided tube using stereo-DIC and single-camera DIC-assisted FPP. (**a**,**b**) Captured images with speckle from left and right cameras in stereo-DIC. (**c**–**e**) Reconstructed disparity map in stereo-DIC using different subset sizes (pixel). (**f**) Texture map before spraying speckle. (**g**) Captured fringe pattern after spraying speckle. (**h**,**i**) Reconstructed phase maps in single-camera DIC-assisted FPP from top and oblique views.
  After spraying speckle pattern, two-camera based stereo-DIC method and single-camera based FPP method are used to measure this complex structure, respectively. The captured images from left and right cameras are shown in Fig. 9a,b and the reconstructed disparity map using different subset sizes are shown in Fig. 9c–e. It is found that the basic outline information is retrieved, but the details in braided straps are blurred by correlation operation. Although reducing subset size can improve the resolution, fine structures cannot be reconstructed even the subset size is reduce to 21 × 21 pixels. For the proposed method, one of the captured fringe patterns is shown in Fig. 9g and the solved phase is shown in Fig. 9h,i. Results show that the fine structures are well-preserved due to the pixel-to-pixel reconstructed ability. Therefore, compared with stereo-DIC, the proposed method can achieve shape measurement with higher resolution and rich details, which supports to further map high-resolution full field defamation and strains on complex parts with fine structure.
3. Application in real-time measurement and detection with less computational cost. It has been proved that the total computational cost of the DIC-assisted FPP system is about half of the traditional stereo-DIC system because half matching calculation is omitted in a single-camera system and the computational cost of phase calculation is much less than that of DIC matching. In previous work, researchers have achieved real-time deformation measurement powered by parallel computation using stereo-DIC system^57,58^ and achieved real-time 3D shape measurement based on FPP system^59^. Therefore, this single-camera system is more effective in real time deformation measurements compared with stereo-DIC and is expected to achieve real-time shape and deformation measurement in assist with GPU. But it should be mentioned that the step size in stereo-DIC method is usually set as half of the subset size to avoid resultant calculation and interpolation algorithm is used to obtain a continuous deformation field in DIC community; pixel-to-pixel calculation is performed in the DIC-assisted FPP system, which will bring extra abundant computational cost in deformation calculation. So, it is suggested that pixel-to-pixel calculation is performed for shape reconstruction but sampling and interpolation is performed for deformation analysis in pursuit of real-time reconstruction.

## Conclusion

In this work, a single-camera system based on DIC-assisted FPP is used to achieve 3D shape measurement and deformation analysis on complex structure parts. Firstly, a measurement system is developed using the presented high-robust and high-efficient 3D shape measurement method based on Gray-coded light, in which full-field 3D geometry of complex structures can be reconstructed pixel-by-pixel. Then, spraying speckle with discrete distribution and median filtering are applied to reduce the influence of speckle on shape measurement; modulation extraction is used to get stable speckle patterns for accurate deformation measurement. And finally, feature-assisted DIC is used to track the matching point at different time and 3D deformation are calculated by comparing the complete 3D shape information of the matching point obtained by FPP. Experimental results have demonstrated the presented method has obvious advantage in reconstructed integrity and computational efficiency compared with the traditional stereo-DIC strategy and it can realize 3D shape and deformation measurement on complex parts, which is challenging and even impossible for the traditional stereo-DIC method.

The proposed measurement method in this work is an extension and complement of the traditional stereo-DIC method for some particular complex structure with deep hole or sharp edges (AM parts, e.g., honeycomb structure) and complex parts with fine structure (polymer composites parts, e.g., biaxially braided composite tube), and it is beneficial to analysis in additive manufacturing, structural mechanics and mechanics of materials.

## Data Availability

Data can be shared upon reasonable request and correspondence should be addressed to Q.Z.
